# Health Preparedness and Narrative Rationality: A Call for Narrative Preparedness

**DOI:** 10.34172/ijhpm.2023.7532

**Published:** 2023-03-05

**Authors:** Eivind Engebretsen, Mona Baker

**Affiliations:** Centre for Sustainable Healthcare Education, Faculty of Medicine, University of Oslo, Oslo, Norway

**Keywords:** Narrative, Public Health, COVID-19, Preparedness, Crisis, Trust

## Abstract

This conceptual paper argues the need for narrative preparedness, understood as the ability to engage and empathize with peoples’ stories and the values they encode, assess them based on the universe in which people live, and acknowledge the narrative rationality of each story – even when it conflicts with the rationality of science. Expanding ‘health preparedness’ to encompass ‘narrative preparedness’ complements the ideals of patient centeredness, which are sometimes betrayed when implemented into concrete decisions because the rationality of science that underpins medical practice fails to make sense of patients’ stories. We outline the central tenets of narrative preparedness and demonstrate its relevance by discussing various responses to mainstream discourses on COVID-19 as a case in point. We discuss and further develop Fisher’s narrative paradigm, which provides a model that complements traditional, scientific rationality with attention to narrative rationality and a radical democratic ground for health political critique. Applying the narrative paradigm to authentic examples of vaccine hesitancy and anti-vaccination demonstrates how closer attention to the way narratives are assessed by different constituencies might help us mitigate some of the sources of resistance and misunderstanding that continue to plague public communication about important medical issues such as pandemics. Health authorities must acknowledge and engage with the stories people believe in and their reasons for doing so. The crucial question for the success of health policy interventions is not only ‘what are the facts’ but ‘how do these facts make sense to people, and why.’ To be prepared for the next pandemic, health professionals must learn to engage with people’s stories and the processes by which they come to be understood and assessed differently by various constituencies.

## Background

**Key Messages**

** Implications for policy makers**

- Alerts health authorities to the importance of narrative preparedness, meaning the capacity among public health authorities and professionals to engage with the various stories about medical crises that people believe in and the values that make them believe in these particular stories.
- Offers policy-makers a model that supports a productive approach to addressing health controversies and communicating medical information in a manner that inspires more trust and speaks to the concerns of different groups in society.
- Promotes an inclusive understanding of health preparedness in order to cope with future pandemics and other health emergencies.
- Provides a tool for addressing epistemic controversies related to future pandemics, and for making sense of the stories people adhere to.
- Outlines the main tenets of a robust theoretical framework consistent with the patient-centred approach that orients modern medical practice and which insists on incorporating the patient’s worldview into medical decision-making.

** Implications for the public**

The paper argues the need and provides a model for engaging with people’s stories and lived experiences in the context of public health emergencies such as pandemics, and explains how policy-makers can use and assess these stories constructively when designing and communicating public health interventions.

There is now strong evidence that countries characterized by high levels of trust – both in government and in various institutions in society, including the medical profession – had lower infection rates and generally performed better during the COVID-19 pandemic than countries where the level of trust was lower.^1,2^ The COVID-19 National Preparedness Collaborators^1^ argue in a paper in the *Lancet* that “if these associations are causal and all countries improved trust in government to the level of Denmark (approximately the 75th percentile of measured countries), … 12.9% fewer global infections would have occurred. Similarly, if all countries improved interpersonal trust to the same level (the 75th percentile of measured countries), the effect would be even larger — 40.3% fewer global infections would have occurred.” Likewise, a recent study that attempted to establish what factors determine acceptance rates of COVID-19 vaccines found that those who expressed trust in their government “were more likely to accept a vaccine than those who said that they did not.”^3^ Countries where levels of acceptance were above 80% were found to be “Asian nations with strong trust in central governments (China, South Korea, and Singapore).”^3^ In France, by contrast, confidence in medical science has declined following various scandals involving the government and drug companies. “The most famous of these,” according to a *Foreign Policy *report which suggests this to be the “real reason France is skeptical of vaccines,” concerns “the diabetes drug Mediator, which was marketed as a weight loss pill and has been linked to the deaths of as many as 2000 people.”^4^

 Still there is limited knowledge about how to engage with the population in a way that inspires trust in situations of crisis. In this paper we argue that health authorities must develop a particular type of social, political and cultural competence to tackle various sources of controversy around medical phenomena such as COVID-19; a type of competence that can provide the basis for a more productive approach to addressing health controversies and communicating medical information in a manner that inspires more trust and speaks to the concerns of different groups in society. We refer to this type of competence as *narrative preparedness,* by which we understand the capacity among public health authorities, as a key institution in society in this context, to engage with the various stories about medical crises that people believe in and, importantly, with the values that make them believe in these particular stories. Unlike the majority of the literature on narratives in the context of policy-making and health communication,^5-8^ we thus focus not on the narratives policy-makers need to elaborate in order to persuade others of the wisdom of adhering to a particular policy, but on the need for policy-makers to listen to and engage with the narratives of diverse communities in order to understand why people refrain from or actively resist abiding by particular policies and how to address this resistance. Like Dillon and Craig,^9^ we do not set out to enable people, including policy-makers, to tell better stories but rather to “empower people to *listen* more critically and more carefully to existing ones.”

 Narrative preparedness must be informed by an approach that is able to analyse and assess competing narratives of the same event on the basis of the values each encodes and, when necessary, contest or adjust these narratives based on the situated principles defined within the narrative itself, as we explain in more detail below.

 It is important to stress at the outset that by narratives we do not mean works of fiction, as is often the case in the scholarly literature on narratives in the medical humanities,^10,11^ nor is our understanding of narrative restricted to genres such as patients’ accounts of illness, as most recently evident in Howell^12^ and is commonly the focus of narrative medicine.^13,14^ Instead, we understand narrative as “the principal and inescapable mode by which we experience the world.”^15^ Rather than a type of discourse, narration must be understood as a type of logic, a fundamental interpretation of the world that is articulated through all forms of discourse and inhabits our thinking. Narrative, as understood here, is not a genre or optional mode of communication. It is not one code among many but a meta-code, “a human universal on the basis of which transcultural messages about the nature of a shared reality can be transmitted.”^16^ This understanding of narrative is shared by numerous scholars of literature,^17^ history,^18^ and sociology,^19^ among others. We choose to draw on the work of Walter Fisher because unlike other scholars of narrative, his narrative paradigm focuses specifically on how we assign value to narratives and on the tension between scientific and narrative rationality. It is particularly helpful, moreover, in offering an explanation of why people make diametrically opposed decisions in relation to a particular issue even when they have access to and agree on the ‘facts’ of a situation. For instance, both white and non-white health professionals receive the same training and have access to the same sources of medical knowledge. But given the long history of structural discrimination that has eroded their confidence in social institutions, a significant proportion of frontline healthcare workers in England (mostly Black and ethnic minorities) turned down the offer of vaccination when it was introduced in early 2021,^20^ despite having access to the same arguments explaining the importance of vaccination, and the same expertise required to assess them, as their white colleagues. Lay members of the public similarly adopt or shun the healthcare options available to them on the basis of how they fit into the narratives to which they subscribe and that constitute their sense of self, rather than on the basis of scientific evidence that they cannot, at any rate, directly assess for themselves. The logic of narrative rationality elaborated by Fisher is attentive to these complexities and “entails a reconceptualization of knowledge, one that permits the possibility of wisdom.”^21^

 Our point of departure, then, is Fisher’s distinction between the world as “a set of logical puzzles that can be solved through appropriate analysis and application of reason conceived as an argumentative construct,” ie, following traditional scientific rationality,^22^ and the world as “a set of stories that must be chosen among to live the good life in a process of continual recreation”^22^; the latter is the domain of narrative rationality. Our claim, moreover, is not that everything we experience comes to us already packaged in narrative form, but rather that we dwell in narratives and that “storytelling is *the *defining feature of humanity,”^23^ implying the being of a certain kind of person with a specific worldview, based on which he or she interprets knowledge and experiences. This has implications for the way we understand scientific rationality and scientific categories, given the assumption that all knowledge – including scientific knowledge – is ultimately configured narratively and can only be processed by the human mind as components in a larger story.^22,24^ Importantly, the concept of narrative rationality suggests that “whatever is taken as a basis for adopting a rhetorical message is inextricably bound to a value – to a conception of the good.”^22^

 This conceptual paper outlines the central tenets of narrative preparedness and demonstrates its relevance by discussing various responses to mainstream discourses on the pandemic, focusing on vaccine hesitancy and anti-vaccination narratives as a case in point. Unlike much work on such narratives, including Larson’s key contribution *Stuck,*^25^ we offer a coherent theoretical framework that goes beyond explaining vaccine hesitancy and the anti-vaccine movement in terms of rumours, misinformation, or conspiracy theories.^26,27^ The examples given all demonstrate different facets of narrative rationality at work and are intended to support our conceptual argument, while acknowledging that a full analysis of vaccine hesitant and anti-vaccination narratives surrounding COVID-19 would require an empirical paper in its own right. Our aim is to stress, from a theoretical point of view, the need to engage actively with narratives that fall outside the scope of scientific rationality and with the ways in which they are assessed by different constituencies, rather than dismissing them as irrational. This, we suggest, can help us mitigate some of the sources of resistance and misunderstanding that continue to plague public communication about important medical issues such as pandemics.

## Capacity to Deal With Controversies

 Much has been written about the importance of health preparedness in the wake of the COVID-19 pandemic. Among other things, COVID-19 has demonstrated the need to explore the root causes of zoonotic transmission through one-health approaches and developing vaccine strategies against zoonotic viruses before the pandemic potential is translated into an actual pandemic.^28-30^ It has also demonstrated the need to improve health surveillance and alert systems at the national, regional and global levels, including the development and application of digital information systems.^31^ The focus of this discussion has not been restricted to scientific and technological issues; social and political aspects of preparedness have also been emphasized, on the basis that health disparities need to be mitigated to build societal resilience and promote sustainable development.^32,33^ According to Haldane and colleagues, “building resilient and equitable societies requires a serious shift in mindsets to engage with and create policies that reflect the broader social, economic, environmental, and political factors in society.”^2^ A recent study that draws on data from Brazil suggests that socioeconomic vulnerabilities have been more important in determining the course of the pandemic than health status, age and other bio-medical risk factors, and that local-level public health responses are crucial to developing health-system resilience and preparedness.^34^ Sireleaf and Clark^31^ similarly argue that COVID-19 has been “a pandemic of inequality” and that future preparedness work should encompass a holistic approach involving partnerships on multiple levels, across government sectors and with groups outside government, in order to address its root causes. A number of studies have further emphasized the need to target health behaviour and belief patterns, among other things by promoting community knowledge and health literacy, as well as appropriate health communication strategies.^35,36^ Paakkari and Okan,^37^ for instance, argue that the COVID-19 ‘infodemic’ has highlighted the poor level of health literacy among various populations, an underestimated aspect of public health preparedness globally.

 All these studies underline the need for adopting an inclusive understanding of health preparedness in order to cope with future pandemics and other health emergencies. Indeed, according to the World Health Organization (WHO) Technical Working Group of the Dynamic Preparedness Metric, preparedness capacity encompasses “all the systems of knowledge, institutions, and infrastructure required to effectively anticipate, mitigate, respond to, and recover from the impact of a health emergency.”^38^ The Independent Panel for Pandemic Preparedness and Response co-chaired by Sireleaf and Clark^31^ similarly concluded that pandemic preparedness requires “sustained whole-of-society efforts” and attention far beyond the health sector. It further underlined the importance of improving risk communication policies and strategies and engaging with the views and stories of various constituencies, including marginalized communities, to build trust and resilience. The COVID-19 National Preparedness Collaborators concluded their large-scale analyses of contextual factors associated with COVID-19 preparedness by emphasizing the need for greater investment in risk communication strategies: “Efforts to improve pandemic preparedness and response for the next pandemic might benefit from greater investment in risk communication and community engagement strategies to boost the confidence that individuals have in public health guidance.”^1^ More specifically, the challenges of risk and crisis communication in terms of tackling scientific uncertainties, rumours and misinformation, and lack of trust in authorities have been emphasized.^35,39,40^ Studies in the field of health communication have underlined the importance of evidence-informed communication strategies^41^ that are tailored to the values of the target audience.^40^ Studies of persuasion^42,43^ and health message design^44,45^ offer frameworks for developing health messages as well as empirical evidence about their effectiveness.^35^ Such theories also include narrative approaches to persuasion.^46,47^ However, as already stated, most of these approaches focus on persuasion and not on the importance of listening to and engaging with stories from the point of view of a critical audience.

 To date, coherent approaches and conceptual models designed to cope with the various sources of concern that people experience in situations of pandemic and that threaten their confidence in public health guidance are few and far between.

## The Narrative Paradigm

 As already mentioned, the model we propose to draw on and extend is Walter Fisher’s narrative paradigm.^22,23^ The basic assumption underpinning this theoretical framework shares our understanding of narrative outlined above, specifically that “[n]o matter how strictly a case is argued – scientifically, philosophically, or legally – it will always be a story, an interpretation of some aspect of the world that is historically and culturally grounded and shaped by human personality.”^22^ Despite the normalizing effect of the narratives we are constantly exposed to and that constitute our social world, we are still capable of reflecting on and questioning these narratives. This is a point we wish to stress and that remains unclear or somewhat downplayed in the original formulation of the narrative paradigm.^23^ We return to it and other limitations of the narrative paradigm later in the article.

 Despite such limitations, Fisher’s narrative paradigm provides us with a basis for understanding how we assess the many competing narratives to which we are constantly exposed. It complements traditional, scientific rationality with attention to narrative rationality. Whereas traditional rationality assesses competing narratives on the basis of the extent to which they follow the rules of logical inference, narrative rationality assesses them on the basis of the extent to which they resonate with the audience’s values and sense of self. The terms Fisher uses to distinguish between these two principles are *probability* (whether the story is coherent and ‘hangs together’) and *fidelity* (whether the story ‘rings true’ and is credible, given the context in which it is elaborated and the lived experience of the characters exposed to it).

 The distinction between traditional and narrative rationality, and between probability and fidelity, explains, for instance, the diametrically opposed responses we have witnessed to scientific arguments about the need to wear a face mask during the COVID-19 crisis. On the one hand, these arguments were vocally rejected by some on the basis that the mandate to wear a mask encroaches on their personal freedom and is a form of control over their bodies; at the same time, others accepted the mandate willingly and saw compliance with it as a matter of moral responsibility to protect themselves and those they may come into contact with. Neither group can simply be dismissed as irrational. The narrative paradigm attempts to make sense of such responses through the concept of narrative rationality outlined above and understood as the way we evaluate the worth of stories based on “good reasons.” Narrative rationality asserts that although the form of an argument and the manner in which it is elaborated do impact its ability to persuade, it is values that are ultimately more persuasive, and these “may be expressed in a variety of modes, of which argument is only one” (emphasis in original).^22^ Greenhalgh makes a similar point in the context of using narrative research in healthcare when she argues that “[s]tories convince not by their objective truth but by their likeness to real life and their emotional impact on the reader or listener.”^48^

 In adopting this approach, our argument is not that all knowledge is equally rational or true, or that any ‘good reason’ is as good as another, but rather that we need to understand how narrative rationality functions in order to prepare effectively for future medical crises. We need to engage with the specific values people adhere to and invest in their narratives in order to understand why they believe in these particular stories and address their concerns. In this sense, the narrative paradigm provides a radical democratic ground for health political critique. It is also democratic in that it refutes the assumption that rationality is a privilege of the few and the exclusive possession of ‘experts’ who (*a*) have specialized knowledge about the issue at hand, (*b*) are cognizant of the argumentative procedures dominant within the field, and (*c*) weigh all arguments in a systematic and deliberative fashion. From the perspective of the narrative paradigm, all human beings are rational. While technical concepts and criteria for judging the rationality of communication can be highly valuable in the specialized contexts in which these concepts are developed, they do not represent absolute standards of truth. To claim that they do is to dismiss large swathes of the population as irrational and incapable of making informed decisions about their own health. This would directly conflict with the patient-centred approach that orients modern medical practice and which insists on incorporating the patient’s worldview into medical decision-making. Indeed, the ideals of patient centeredness are sometimes betrayed when implemented into concrete decisions because the rationality of science that underpins medical practice fails to make sense of the patients’ stories.^49^ A democratic understanding of rationality along the lines elaborated in the narrative paradigm, we argue, is a prerequisite to elaborating effective narratives that can enhance the reception of medical knowledge and reduce some of the sources of resistance and misunderstanding that continue to plague public communication during critical events such as pandemics.

 Experts themselves, too, are not immune to the workings of narrative rationality. Once the expert moves out of the immediate context in which technical knowledge is assessed from a scientific perspective, and into the complex, messy space of everyday life, he or she becomes subject to the demands of narrative rationality. When the medical expert, for instance, engages in public discourse regarding pandemic-related measures or in dialogue with patients about everyday health problems, he or she is obliged to leave the rationality of their technical community behind and submit to what are ultimately narrative criteria for deciding which of a range of competing narratives they are exposed to at any specific time is most worthy of believing and adhering to. A striking example comes from the controversy surrounding the AstraZeneca vaccine in 2021. Speaking on CTV on May 4, 2021, Dr. Quanch-Thanh, Chair of the National Advisory Committee on Immunization in Canada, controversially admitted that risk cannot necessarily be calculated rationally: “If, for instance, my sister was to get the AstraZeneca vaccine and die of a thrombosis when I know that it could have been prevented and that she’s not in a high-risk area, I’m not sure I could live with it.”^50^ She was later criticized for fuelling fear and hesitancy through her statement. On an epistemological level, however, her unguarded response reveals the extent to which medical discourses depend on narrative rationality but at the same time struggle to make sense of it. While trying to defend, from the point of view of scientific rationality, the National Advisory Committee on Immunization’s decision to advise young people to wait for the preferred vaccine, she inadvertently admitted that what ultimately matters in practice is whether the decision to take or not take a specific vaccine is consistent with – speaks to – people’s lived experience and its potential risk to loved ones, rather than its overall risk assessment. This is about whether a person embedded in space and time and emotionally connected to others can “live with” a particular decision they have to make, not about assessments of risk in the disconnected and sanitized environment of the lab.^23^

 Narrative probability and fidelity may be thought of as *tests* that we apply – whether instinctively or through conscious reasoning – to decide whether a narrative coheres and offers good reasons for action and belief. A message that is judged by a particular audience to be high in narrative probability and narrative fidelity enhances identification and is more likely to be adopted or adhered to by members of that audience. Understanding these ‘tests,’ as demonstrated below, can create awareness about the multiple and conflicting stories in which the COVID-19 controversies were situated and the values that made people accept or reject particular stories. Fisher’s model also provides a tool for addressing epistemic controversies related to future pandemics, and for making sense of the stories people adhere to, or not adhere to, and their reasons for doing so.

## Narrative Probability (Coherence)

 Narrative probability or coherence concerns the internal and external consistency and integrity of a narrative. It is assessed on the basis of three considerations that are all familiar components of traditional reasoning: first, the structural makeup of the narrative, or the way it coheres internally (*structural or argumentative coherence*); second, its external consistency and completeness in terms of how it differs from or accords with other stories on the same issue that we are aware of (*material coherence*); and third, its believability in terms of *who* is telling it and the extent to which we can trust them (*characterological coherence*) – whether they are real life narrators such as WHO, or characters featured in a narrative (fictional or otherwise) told by a real life narrator we associate with certain qualities.

 During the pandemic, assessment by different groups of the extent to which official public health narratives adhered to these different forms of coherence and integrity influenced the way they responded to them. The debate about vaccines in particular highlighted the fact that there are divergent views within the scientific community itself on when new evidence may be ready to be put into political action, and what considerations – other than the findings of randomized controlled trials – might be brought to bear on the decision. This complicated the process of assessing narratives about new vaccines in terms of both their structural and material coherence. A good example of **structural incoherence** comes from a series of statements on various vaccines issued by the European Medicine Agency (EMA) following the first instances of reported blood clots in Denmark and Norway. On March 11, 2021, EMA declared that “there is currently no indication that vaccination has caused these conditions, which are not listed as side effects with this vaccine.”^51^ A little less than a month later, the EMA’s safety committee concluded that “unusual blood clots with low blood platelets should be listed as very rare side effects of Vaxzevria (formerly COVID-19 Vaccine AstraZeneca).”^52^ In a statement intended for health professionals issued on the same date (April 7, 2021), EMA explicitly stated that “*a causal relationship* between the vaccination with Vaxzevria and the occurrence of thrombosis in combination with thrombocytopenia is considered plausible” (emphasis added).^53^ An updated statement on 20 May makes no mention of the “causal relationship” and instead presents the connection between the two as a mere observation: “A combination of thrombosis and thrombocytopenia, in some cases accompanied by bleeding, *has been observed very rarely* following vaccination with Vaxzevria” (emphasis added).^54^

 In terms of **material incoherence**, the haste with which a solution had to be found to arrest the spread of the disease, and the pressure on the medical community to produce a miracle cure, both resulted in widespread discussions about studies drawing conclusions that are premature or even fraudulent,^55,56^ making it difficult for non-experts to decide who or what to believe as different sources made different, conflicting claims and as trust in the medical profession was gradually eroded by such reports.

 Another example of the way **material incoherence** continues to impact the uptake of vaccines is the belief that they involve some form of genetic modification. The idea that gene therapy is unnatural and ‘anti-human’ is far from new. Anti-GMO (genetically modified organism) activists have drawn since the late 1980s on narratives that link GMOs with pollution, contamination or monstrousness. Similar narratives have also been used by politicians, especially in Europe, where the legislation on GMOs is particularly restrictive. As Christiansen et al^57^ have pointed out, the restrictive rules imposed on GMOs are fundamentally based on the value of naturalness, since the organisms covered by the legislation are those ‘‘in which the genetic material has been altered in a way that does not occur naturally by mating and/or natural recombination,’’ according to the European Commission’s archived page on Biotechnology.^58^ In a blog post on *The Daily Beast *that questions this rationality and the values that underpin it, Anslow thus concludes that “In this pandemic anti-vaxxers did not need to discredit 200 years of vaccine efficacy, or explain away scientific consensus. They just needed to sow doubt about emerging biotechnologies, a job that had already been largely done for them by the press and politicians. Biotechnophobia was already endemic.”^59^ This critique of the structurally and matterially incoherent attitudes of European politicians with regard to gene engineering is echoed by Brooks, an agricultural economist, who argues in a blog post on Open Access Government that European politicians show inconsistency when they queue up to praise the breakthroughs of the new vaccines^60^:

 “These vaccines use the very same techniques of genetic modification or gene editing that most European politicians have spent the last 25 years preventing their citizens and farmers from having access to for the production and consumption of food, feed and fibre crops and which so-called environmental advocacy groups have opposed unequivocally.”

 “If these politicians and advocacy groups were being consistent with their past behaviour, they would be vigorously campaigning against these vaccines’ approval and publicly stating that they personally will not be using them.”

 Assessment of **characterological coherence** does not only apply to individuals but also to institutions, including the political and medical institutions in society. In the case of vaccines and other pharmaceutical interventions, characterological coherence seems to work in complex ways that are influenced by centuries of public opposition to vaccination, and by repeated attempts on the part of governments to suppress this opposition by passing laws that make certain types of vaccines mandatory. Alongside these legal measures, institutions representing medical practitioners also have a history of censuring doctors who act in ways that undermine specific vaccination campaigns. In the context of COVID-19, a recent example is Dr. Gerard Waters, who was suspended from the medical register by the High Court of Ireland in April 2021 for refusing to vaccinate his patients against COVID-19.^61^ Dr. Waters, who believed the vaccine to be “untrustworthy and unnecessary”^62^ and “disagreed with how quickly the vaccines had been developed,”^63^ described himself as a ‘conscientious objector,’ thus invoking associations with pacifism and the Christian principle ‘thou shalt not kill,’ used by the Quakers in particular to justify refusal of armed service in both World Wars. The framing of a narrative such as Dr. Waters’s is important in influencing assessments of characterological coherence. In this case, powerful institutions are narrated as exercising their superior power against a principled individual who holds fast to his beliefs despite the adverse consequences to his career. This type of storyline appeals to particular values that many people hold dear, such as courage and integrity, which can provide ‘good reasons’ for believing dissenting rather than official, mainstream characters.

 Narrative preparedness requires us to acknowledge, analyze and address such instances of (in)coherence at all levels of government and various sectors of the medical community if we are to enhance the reception of and adherence to medical advice and ultimately improve health outcomes.

## Narrative Fidelity

 Whereas narrative probability involves logical inference and traditional modes of argumentation, narrative fidelity is about how we assess the truth qualities of a story, which we can only do (according to Fisher) in terms of how well it resonates with our experience of the world, and the experience of those we have reason to trust. The operative principle of narrative fidelity is therefore “*identification* rather than deliberation.”^22^ Identification does not mean we necessarily have to *share* the experiences of protagonists to find the story in which they are depicted credible: it merely requires that these protagonists’ experiences appear to us to be “true to life – in principle.”^22^ In other words, we identify to the extent that we can imagine ourselves as characters in a given story and accept that had we been these characters our experiences would probably have been similar. It is this type of identification that makes empathy possible even in the case of narratives that depict protagonists such as civilians caught up in war or starving children in poorer countries, whose circumstances are far removed from our own.

 Narrative fidelity ultimately rests on an assessment of transcendental values. These are values such as liberty or honesty that we tend to take for granted and are rarely the subject of dispute. Transcendental values often exceed everyday values such as precision and accuracy in the context of scholarly work. They may also exceed pragmatic values such as efficiency and success. The ultimate values we live by “look not only to the past and present, but also to the future, the future beyond the immediate moment”; they include “justice, happiness, and humanity,” but for Fisher the ultimate value is “love, that is an abiding concern for the welfare and well-being of others.”^22^

 A transcendental value that featured prominently in numerous protests against various measures such as lockdowns and vaccination in the context of COVID-19 is freedom, one’s own and that of others: freedom of movement, of religious practice, of choice (including choice to decide what to do with one’s body), among other things. Several campaigns featured ‘freedom’ in their titles, as in the Freedom Convoy protests in Ottawa (February 2022), or were organized by groups which explicitly campaigned under the banner of ‘freedom’ (the UK Freedom Movement and Unite for Freedom, for example). Such movements garnered considerable support precisely because they appealed to a value that most of us share and consider sacrosanct – under most circumstances. It is no surprise, therefore, that a January 2021 survey^3^ of potential acceptance of a COVID-19 vaccine that involved 13 426 people in 19 countries found “a discrepancy between reported acceptance of a COVID-19 vaccine and acceptance if vaccination was mandated by one’s employer.” All respondents to the survey, “regardless of nationality” and despite marked differences in the level of vaccine acceptance across countries, “reported that they would be less likely to accept a COVID-19 vaccine if it were mandated by employers.” The authors conclude that “[t]his finding across all countries with both high and low reported vaccine acceptance proportions suggests that promoting voluntary acceptance is a better option for employers.”^3^ The implication for health policy is clear. The fact that freedom is a transcendental value, is sacrosanct, for so many people means that the more intrusive and severe the measures adopted to control individual behaviour, the more likely it is for increasing numbers of people to react negatively to the intrusion into their personal lives, and – importantly – into those of others, irrespective of their own position on the subject of intrusion and their assessment of the facts of the situation.

 Narrative fidelity is also assessed in terms of whether or not a story resonates with our experience of the world, and this is particularly clear in the case of racial and other types of discrimination. Several studies have suggested that racial bias is endemic in the medical field. To cite just one example, Hoffman et al^64^ found that white medical students “believe that the black body is biologically different” and that black people feel less pain than white people, and hence tend to under-prescribe pain relief medication for them. Reasons for the persistent distrust in health institutions among Black and other minority populations is also rooted in history. Yearby et al^65^ maintain that as far back as the Jim Crow era (1875-1968), “racism has implicitly and explicitly been an integral part of the US government’s structuring and financing of the healthcare system,” which routinely provides inferior facilities and coverage to the black population. An extreme example of the kind of systemic racism this part of the population has suffered is the so-called Tuskegee Syphilis Study, which was conducted between 1932 and 1972 by the United States Public Health Service and the Centers for Disease Control and Prevention on a group of nearly 400 African Americans with syphilis. Participants were told that they were receiving free medical care; in fact, they were merely being observed for a study of untreated syphilis. Dozens died as a result. Thus recent studies now acknowledge that the fact that vaccine hesitancy is particularly common among the Black community is “likely grounded in a long history of structurally racist systems which have led to health inequalities and injustices.”^66^

 Even though a given black person may never have been subjected to the kind of extreme racism demonstrated in the Tuskegee Syphilis Study, their exposure to this narrative will still make them apprehensive about health authorities and institutions because they will see themselves as part of that group and hence a potential target of discrimination. Morgan^67^ shows how this kind of reasoning influenced the response among people from Black, Asian and minority ethnic groups when the medical authorities proposed starting the vaccine roll out with the most vulnerable communities during the first wave of the pandemic:

 “This caused concern among these communities, because they are not normally at the front of the queue when it comes to the best medical treatments, particularly those in lower socioeconomic classes. Some people began to speculate that it was because it was an experimental vaccine and Black people were being used as guinea pigs.”

 Morgan concludes that for some, “this will have triggered alarm bells and brought up the many historical examples of Black people being used for experimental or unethical medical treatments.”^67^

 Against this background, narrative preparedness requires us to develop an ability to understand and empathize with other peoples’ stories and the values they encode, to assess these stories based on the universe in which these people live and operate and to acknowledge the narrative fidelity of their stories – even when these are in conflict with the rationality of science.

## The Narrative Paradigm Revisited

 Fisher’s narrative paradigm is not without its critics and limitations. Warnick,^68^ for example, has argued that Fisher’s theory is based on a simplified understanding of the rational logic that he refutes. She claims that Fisher only attacks one subform of what he calls traditional rationality – technical rationality – without acknowledging other forms, such as practical reasoning and moral judgement. Furthermore, while acknowledging that people can be wrong, he is silent on how they can avoid being deluded, given his dismissal of traditional rationality. As she puts it, “a rhetorical narrative may ‘ring true’ in the lives of particular audience members, may resonate with their own experience and that of those who they admire, and nevertheless be a bad story.” The success of Nazism is just one example. Acknowledging this criticism, we do not suggest that everything that ‘rings true’ to an audience is necessarily good according to some universal standard. We only claim that any argument is inevitably assessed as a story and with reference to specific values that the audience invest in and consider central to the way they wish to live their lives. Importantly, we diverge from Fisher in incorporating into our revised model the belief that an audience is capable of entertaining novel narratives that do not immediately resonate with their existing values and sense of self, and “acknowledging the importance of opening people’s minds to ‘creative possibilities’ that they may not be alert to.”^23^

 As emphasized in the studies cited in the introduction, effective public health measures are strongly linked to communication and persuasion, in that efforts to change behaviour are necessarily communicative acts. In order to inspire trust and adherence, health authorities must acknowledge and engage with stories like those we have documented above. The concerns of those who object to various restrictions such as wearing face masks or who have concerns about vaccination can only be addressed and contested by understanding and engaging with the logics of the stories to which they subscribe. Despite these limitations of the narrative paradigm as acknowledged above, we believe that Fisher’s model can help throw light on some of the blind spots of the dominant epistemic paradigm in public health and, even more importantly, offer tools that can help us be better prepared to face the anxieties and concerns that will continue to plague our responses to future pandemics unless we learn to address them more effectively. The crucial question for the success of health policy interventions is not only ‘what are the facts’ but ‘how do these facts make sense to people, and why.’^22^ This does not mean that establishing and communicating scientific facts is not essential to successful public health work. Rather, it means that we do not get anywhere with science unless it makes sense to people, ie, unless scientific facts are presented in a manner that either resonates with people’s current values and experiences or is capable of alerting them to new possibilities they can potentially make sense of and buy into. Facts cannot make sense in a vacuum: they only make sense as stories that reinforce or productively challenge the narratives that make up our existing moral universe.

 While facts are the cornerstone of the rational world paradigm, which proceeds by considering “whether the statements in a message that purport to be ‘facts’ are indeed ‘facts,’”^15^ the narrative paradigm considers all facts to be value-laden and assumes that assessing whatever is presented as fact always involves considering “the explicit or implicit values embedded in a message.”^15^ Writing in *The Conversation* in July 2021, Manuel León Urrutia^69^ draws attention to how COVID-19 data have proved to be complex and changeable. As an expert in data literacy, he reflects on how the visibility of data “has assumed a central role in determining the degree of society’s freedom since March 2020.” Highly specialist statistical jargon and data visualizations now pervade public discourse about the pandemic. But as the author argues, increased knowledge of specialized terms such as ‘flattening the curve’ do not necessarily contribute to better understanding, and even less to increased consensus about the need for various types of intervention. On the contrary, “this data deluge can contribute to the polarisation of public discourse” rather than resolving controversies. Although data “is supposed to be objective and empirical,” Urrutia argues, “it has assumed a political, subjective hue during the pandemic.” This is understandable given that people can only make sense of data by incorporating it into larger narratives of the pandemic. It means that rather than trying to resolve controversies by providing more data, which is the standard public health approach, health authorities need to engage more actively with people’s values and experiences – ie, with the stories that circulate in our communities.

 Ultimately, we maintain, it is through narratives that knowledge about medical and other phenomena is communicated to others, enters the public space, and provokes discussion and disagreements. To be prepared for the next pandemic, health professionals should therefore be equipped to deal with people’s stories and engage with the processes by which they come to be understood and assessed differently by various constituencies.

## Acknowledgements

 This article was written as part of the international research program on “The Body in Translation: Historicising and Reinventing Medical Humanities and Knowledge Translation” at the Centre for Advanced Study at the Norwegian Academy of Science and Letters in Oslo during the academic year 2019/2020.

## Ethical issues

 This is a conceptual paper and no ethical approval is needed.

## Competing interests

 Authors declare that they have no competing interests.

## Authors’ contributions

 Both authors contributed equally to the design and drafting of the manuscript. Both authors have approved the final version.

## Funding

 This work was supported by the Centre for Advanced Study at the Norwegian Academy of Science and Letters as part of the research project “The Body in Translation: Historicising and Reinventing Medical Humanities and Knowledge Translation” [no grant number] and by Centre for Sustainable Healthcare Education, University of Oslo, Norway [no grant number].
